# Abandoning the use of tension in tibial fracture nailing is associated with lower rate for acute compartment syndrome?

**DOI:** 10.1186/s10195-024-00780-4

**Published:** 2024-09-09

**Authors:** Ville Ponkilainen, Heikki Nurmi

**Affiliations:** Central Finland Central Hospital Nova, Jyväskylä, Finland

Dear Editor,

We recently read the article by Honkonen et al. [1] with great interest, as it presented groundbreaking results that could significantly impact the management of tibial shaft fractures, particularly in the context of avoiding unnecessary fasciotomies. However, upon closer examination, we identified a potential confounding bias that warrants recognition.

In their introduction, Honkonen et al. acknowledge previous evidence indicating that calcaneal traction can lead to increased postoperative intracompartmental pressure (ICP) in tibial fractures during intramedullary nailing (IMN) [2]. They also suggest that traction, along with the flexed position of the knee and popliteal support used in the infrapatellar (IP) IMN technique, are considered the main perioperative risk factors for the development of acute compartment syndrome (ACS). However, they fail to discuss the mechanisms by which different approaches (suprapatellar versus infrapatellar) might affect the risk of ACS.

The correlation between traction and increased ICP was previously identified by Shakespeare and Henderson in 1982 [3]. They concluded that for every kilogram of traction applied, the deep posterior pressure rose by 5.7% and the anterior pressure by 1.6%. However, to our knowledge, no studies have explained the bioplausibility behind the correlation between IP IMN and ACS, nor have any studies demonstrated the risk of ACS if both approaches were conducted without traction. This raises the question: is the real reason behind higher ACS rates truly the IP approach, or is it the use of traction?

In the results section, the authors do not describe the most relevant data: the position in which IP IMN was conducted. In the discussion, they mention that all IP IMNs were conducted with traction at that time. Therefore, the results of this study provide more insight into the correlation between the use of traction and ACS than the correlation between SP and IP approaches. As IP IMN can be conducted without traction, the authors should not conclude that the approach is the main issue, but rather the traction itself. Thus, we find it misleading that the title and conclusions focus on the confounder, the approach, rather than traction, despite previous evidence indicating that traction is a risk factor for ACS.

In conclusion, while we appreciate the valuable contribution of Honkonen et al.’s study, we believe that the comprehensive discussion should focus on the role of traction in ACS development rather than the approach. We hope that future studies will address this issue to provide a clearer understanding of the factors contributing to ACS in tibial shaft fractures.

## Data Availability

Not applicable.

## References

[1] Honkonen EE, Repo JP, Lehtokangas H, Luoma E, Uimonen M, Nurmi S et al (2024) Suprapatellar tibial fracture nailing is associated with lower rate for acute compartment syndrome and the need for fasciotomy compared with the infrapatellar approach. J Orthop Traumatol 25(1):5 38282098 10.1186/s10195-024-00749-3 PMC10822828

[2] Kutty S, Laing AJ, Prasad CVR, McCabe JP (2005) The effect of traction on compartment pressures during intramedullary nailing of tibial-shaft fractures: a prospective randomised trial. Int Orthop 29(3):186–190 15815904 10.1007/s00264-005-0651-9 PMC3456878

[3] Shakespeare D, Henderson N (1982) Compartmental pressure changes during calcaneal traction in tibial fractures. J Bone Joint Surg Br Vol 64(4):498–499 10.1302/0301-620X.64B4.7096431 7096431

